# The Psychological Science Accelerator’s COVID-19 rapid-response dataset

**DOI:** 10.1038/s41597-022-01811-7

**Published:** 2023-02-11

**Authors:** Erin M. Buchanan, Savannah C. Lewis, Bastien Paris, Patrick S. Forscher, Jeffrey M. Pavlacic, Julie E. Beshears, Shira Meir Drexler, Amélie Gourdon-Kanhukamwe, Peter R Mallik, Miguel Alejandro A. Silan, Jeremy K. Miller, Hans IJzerman, Hannah Moshontz, Jennifer L. Beaudry, Jordan W. Suchow, Christopher R. Chartier, Nicholas A. Coles, MohammadHasan Sharifian, Anna Louise Todsen, Carmel A. Levitan, Flávio Azevedo, Nicole Legate, Blake Heller, Alexander J. Rothman, Charles A. Dorison, Brian P. Gill, Ke Wang, Vaughan W. Rees, Nancy Gibbs, Amit Goldenberg, Thuy-vy Thi Nguyen, James J. Gross, Gwenaêl Kaminski, Claudia C. von Bastian, Mariola Paruzel-Czachura, Farnaz Mosannenzadeh, Soufian Azouaghe, Alexandre Bran, Susana Ruiz-Fernandez, Anabela Caetano Santos, Niv Reggev, Janis H. Zickfeld, Handan Akkas, Myrto Pantazi, Ivan Ropovik, Max Korbmacher, Patrícia Arriaga, Biljana Gjoneska, Lara Warmelink, Sara G. Alves, Gabriel Lins de Holanda Coelho, Stefan Stieger, Vidar Schei, Paul H. P. Hanel, Barnabas Szaszi, Maksim Fedotov, Jan Antfolk, Gabriela-Mariana Marcu, Jana Schrötter, Jonas R. Kunst, Sandra J. Geiger, Adeyemi Adetula, Halil Emre Kocalar, Julita Kielińska, Pavol Kačmár, Ahmed Bokkour, Oscar J. Galindo-Caballero, Ikhlas Djamai, Sara Johanna Pöntinen, Bamikole Emmanuel AGESIN, Teodor Jernsäther, Anum Urooj, Nikolay R. Rachev, Maria Koptjevskaja-Tamm, Murathan Kurfalı, Ilse L. Pit, Ranran Li, Sami Çoksan, Dmitrii Dubrov, Tamar Elise Paltrow, Gabriel Baník, Tatiana Korobova, Anna Studzinska, Xiaoming Jiang, John Jamir Benzon R. Aruta, Jáchym Vintr, Faith Chiu, Lada Kaliska, Jana B. Berkessel, Murat Tümer, Sara Morales-Izquierdo, Hu Chuan-Peng, Kevin Vezirian, Anna Dalla Rosa, Olga Bialobrzeska, Martin R. Vasilev, Julia Beitner, Ondřej Kácha, Barbara Žuro, Minja Westerlund, Mina Nedelcheva-Datsova, Andrej Findor, Dajana Krupić, Marta Kowal, Adrian Dahl Askelund, Razieh Pourafshari, Jasna Milošević Đorđević, Nadya-Daniela Schmidt, Ekaterina Baklanova, Anna Szala, Ilya Zakharov, Marek A. Vranka, Keiko Ihaya, Caterina Grano, Nicola Cellini, Michał Białek, Lisa Anton-Boicuk, Ilker Dalgar, Arca Adıgüzel, Jeroen P. H. Verharen, Princess Lovella G. Maturan, Angelos P. Kassianos, Raquel Oliveira, Martin Čadek, Vera Cubela Adoric, Asil Ali Özdoğru, Therese E. Sverdrup, Balazs Aczel, Danilo Zambrano, Afroja Ahmed, Christian K. Tamnes, Yuki Yamada, Leonhard Volz, Naoyuki Sunami, Lilian Suter, Luc Vieira, Agata Groyecka-Bernard, Julia Arhondis Kamburidis, Ulf-Dietrich Reips, Mikayel Harutyunyan, Gabriel Agboola Adetula, Tara Bulut Allred, Krystian Barzykowski, Benedict G Antazo, Andras N. Zsido, Dušana Dušan Šakan, Wilson Cyrus-Lai, Lina Pernilla Ahlgren, Matej Hruška, Diego Vega, Efisio Manunta, Aviv Mokady, Mariagrazia Capizzi, Marcel Martončik, Nicolas Say, Katarzyna Filip, Roosevelt Vilar, Karolina Staniaszek, Milica Vdovic, Matus Adamkovic, Niklas Johannes, Nandor Hajdu, Noga Cohen, Clara Overkott, Dino Krupić, Barbora Hubena, Gustav Nilsonne, Giovanna Mioni, Claudio Singh Solorzano, Tatsunori Ishii, Zhang Chen, Elizaveta Kushnir, Cemre Karaarslan, Rafael R. Ribeiro, Ahmed Khaoudi, Małgorzata Kossowska, Jozef Bavolar, Karlijn Hoyer, Marta Roczniewska, Alper Karababa, Maja Becker, Renan P. Monteiro, Yoshihiko Kunisato, Irem Metin-Orta, Sylwia Adamus, Luca Kozma, Gabriela Czarnek, Artur Domurat, Eva Štrukelj, Daniela Serrato Alvarez, Michal Parzuchowski, Sébastien Massoni, Johanna Czamanski-Cohen, Ekaterina Pronizius, Fany Muchembled, Kevin van Schie, Aslı Saçaklı, Evgeniya Hristova, Anna O. Kuzminska, Abdelilah Charyate, Gijsbert Bijlstra, Reza Afhami, Nadyanna M. Majeed, Erica D. Musser, Miroslav Sirota, Robert M. Ross, Siu Kit Yeung, Marietta Papadatou-Pastou, Francesco Foroni, Inês A. T. Almeida, Dmitry Grigoryev, David M. G. Lewis, Dawn L. Holford, Steve M. J. Janssen, Srinivasan Tatachari, Carlota Batres, Jonas K. Olofsson, Shimrit Daches, Anabel Belaus, Gerit Pfuhl, Nadia Sarai Corral-Frias, Daniela Sousa, Jan Philipp Röer, Peder Mortvedt Isager, Hendrik Godbersen, Radoslaw B. Walczak, Natalia Van Doren, Dongning Ren, Tripat Gill, Martin Voracek, Lisa M. DeBruine, Michele Anne, Sanja Batić Očovaj, Andrew G. Thomas, Alexios Arvanitis, Thomas Ostermann, Kelly Wolfe, Nwadiogo Chisom Arinze, Carsten Bundt, Claus Lamm, Robert J Calin-Jageman, William E. Davis, Maria Karekla, Saša Zorjan, Lisa M. Jaremka, Jim Uttley, Monika Hricova, Monica A Koehn, Natalia Kiselnikova, Hui Bai, Anthony J. Krafnick, Busra Bahar Balci, Tonia Ballantyne, Samuel Lins, Zahir Vally, Celia Esteban-Serna, Kathleen Schmidt, Paulo Manuel L. Macapagal, Paulina Szwed, Przemysław Marcin Zdybek, David Moreau, W. Matthew Collins, Jennifer A. Joy-Gaba, Iris Vilares, Ulrich S. Tran, Jordane Boudesseul, Nihan Albayrak-Aydemir, Barnaby James Wyld Dixson, Jennifer T Perillo, Ana Ferreira, Erin C. Westgate, Christopher L. Aberson, Azuka Ikechukwu Arinze, Bastian Jaeger, Muhammad Mussaffa Butt, Jaime R. Silva, Daniel Shafik Storage, Allison P Janak, William Jiménez-Leal, Jose A. Soto, Agnieszka Sorokowska, Randy McCarthy, Alexa M Tullett, Martha Frias-Armenta, Matheus Fernando Felix Ribeiro, Andree Hartanto, Paul A. G. Forbes, Megan L. Willis, María del Carmen Tejada R, Adriana Julieth Olaya Torres, Ian D Stephen, David C. Vaidis, Anabel de la Rosa-Gómez, Karen Yu, Clare A. M. Sutherland, Mathi Manavalan, Behzad Behzadnia, Jan Urban, Ernest Baskin, Joseph P. McFall, Chisom Esther Ogbonnaya, Cynthia H. Y. Fu, Rima-Maria Rahal, Izuchukwu L. G. Ndukaihe, Thomas J. Hostler, Heather Barry Kappes, Piotr Sorokowski, Meetu Khosla, Ljiljana B. Lazarevic, Luis Eudave, Johannes K. Vilsmeier, Elkin O. Luis, Rafał Muda, Elena Agadullina, Rodrigo A. Cárcamo, Crystal Reeck, Gulnaz Anjum, Mónica Camila Toro Venegas, Michal Misiak, Richard M. Ryan, Nora L. Nock, Giovanni A. Travaglino, Michael C. Mensink, Gilad Feldman, Aaron L. Wichman, Weilun Chou, Ignazio Ziano, Martin Seehuus, William J. Chopik, Franki Y. H. Kung, Joelle Carpentier, Leigh Ann Vaughn, Hongfei Du, Qinyu Xiao, Tiago J. S. Lima, Chris Noone, Sandersan Onie, Frederick Verbruggen, Theda Radtke, Maximilian A. Primbs

**Affiliations:** 1 Analytics, Harrisburg University of Science and Technology, Harrisburg, USA; 2 Ashland University, Ashland, USA; 3 Université Grenoble Alpes, Grenoble, France; 4 Busara Center for Behavioral Economics, Nairobi, Kenya; 5 Department of Psychology, University of Mississippi, Oxford, USA; 6 Alliant International University, San Diego, USA; 7 Department of Neurology, Mauritius Hospital Meerbusch, Meerbusch, Germany; 8 King’s College, London, United Kingdom; 9 Kingston University, London, United Kingdom; 10 Hubbard Decision Research, Glen Ellyn, USA; 11 Annecy Behavioral Science Lab, Université Lumière Lyon 2, Lyon, France; 12 Willamette University, Salem, USA; 13 LIP/PC2S, Université Grenoble Alpes, Grenoble, France; 14 Institut Universitaire de France, Paris, France; 15 University of Wisconsin - Madison, Madison, USA; 16 Department of Psychological Sciences, Swinburne University of Technology, Melbourne, Australia; 17 School of Business, Stevens Institute of Technology, Hoboken, USA; 18 Center for the Study of Language and Information, Stanford University, Stanford, USA; 19 Department of Psychology, University of Tehran, Tehran, Iran; 20 School of Psychology and Neuroscience, University of St Andrews, St Andrews, United Kingdom; 21 Occidental College, Los Angeles, USA; 22 Department of Psychology, University of Cambridge, Cambridge, United Kingdom; 23 Illinois Institute of Technology, Chicago, USA; 24 Hobby School of Public Affairs, University of Houston, Houston, USA; 25 University of Minnesota, Minneapolis, USA; 26 Kellogg School of Management, Evanston, USA; 27 Mathematica, Princeton, USA; 28 Harvard University, Boston, USA; 29 Department of Social and Behavioral Sciences, Harvard T.H. Chan School of Public Health, Boston, USA; 30 Harvard Kennedy School, Boston, USA; 31 Harvard Business School, Boston, USA; 32 Durham University, Durham, United Kingdom; 33 Department of Psychology, Stanford University, Stanford, CA USA; 34 CLLE, Université de Toulouse, Toulouse, France; 35 University of Sheffield, Sheffield, United Kingdom; 36 Institute of Psychology, University of Silesia in Katowice, Katowice, Spain; 37 Penn Center for Neuroaesthetics, ChatLab, University of Pennsylvania, Philadelphia, US; 38 Radboud University, Nijmegen, Netherlands; 39 Department of Psychology, Mohammed V University, Rabat, Morocco; 40 Université Paris Cité, Paris, France; 41 FOM University of Applied Sciences, Essen, Germany; 42 Department of Education, Social Sciences and Humanities, Faculty of Human Kinetics, University of Lisbon, Lisbon, Portugal; 43 Environmental Health Institute, Faculty of Medicine, University of Lisbon, Lisbon, Portugal; 44 Department of Psychology and School of Brain Sciences and Cognition, Ben Gurion University, Beersheba, Israel; 45 Department of Management, Aarhus University, Aarhus, Denmark; 46 MIS Department, Ankara Science University, Ankara, Turkey; 47 Center for Social and Cultural Psychology, Université libre de Bruxelles, Brussels, Belgium; 48 Faculty of Education, Institute for Research and Development of Education, Charles University, Prague, Czechia; 49 Faculty of Education, University of Presov, Presov, Slovakia; 50 Western Norway University of Applied Sciences, Bergen, Norway; 51 CIS_Iscte, ISCTE - University Institute of Lisbon, Lisbon, Portugal; 52 Macedonian Academy of Sciences and Arts, Skopje, North Macedonia; 53 Lancaster University, Lancaster, United Kingdom; 54 Center for Psychology at University of Porto, Faculty of Psychology and Educational Sciences, University of Porto, Porto, Portugal; 55 University College Cork, Cork, Ireland; 56 Department of Psychology and Psychodynamics, Karl Landsteiner University of Health Sciences, Krems an der Donau, Austria; 57 Department of Strategy and Management, NHH Norwegian School of Economics, Bergen, Norway; 58 University of Essex, Essex, United Kingdom; 59 Institute of Psychology, ELTE - Eötvös Loránd University, Budapest, Hungary; 60 Institute for Linguistic Studies, Russian Academy of Sciences, Moscow, Russia; 61 Faculty of Arts, Psychology and Theology, Åbo Akademi University, Turku, Finland; 62 Department of Psychology, “Lucian Blaga” University of Sibiu, Sibiu, Romania; 63 Department of Psychiatry, Medical Faculty, Pavol Jozef Šafárik University in Košice, Košice, Slovakia; 64 Department of Psychology, University of Oslo, Oslo, Norway; 65 Environmental Psychology, Department of Cognition, Emotion, and Methods, Faculty of Psychology, University of Vienna, Vienna, Austria; 66 Alex Ekwueme Federal University, Ndufu-Alike, Nigeria; 67 Department of Psychological Counseling and Guidance, Muğla Sıtkı Koçman University, Kotekli, Turkey; 68 Jan Dlugosz University in Czestochowa, Czestochowa, Poland; 69 Department of Psychology, Faculty of Arts, Pavol Jozef Šafárik University in Košice, Košice, Slovakia; 70 Mohammed V University, Rabat, Morocco; 71 Facultad de Ciencias Sociales y Humanas, Universidad Manuela Beltrán, Bogota, Colombia; 72 Department of Pure & Applied Psychology, Adekunle Ajasin University, Akungba Akoko, Nigeria; 73 Department of Psychology, Stockholm University, Stockholm, Sweden; 74 La Trobe University, Melbourne, Australia; 75 Department of General, Experimental, Developmental, and Health Psychology, Sofia University St. Kliment Ohridski, Sofia, Bulgaria; 76 Department of Linguistics, Stockholm University, Stockholm, Sweden; 77 Institute of Human Sciences, University of Oxford, Oxford, United Kingdom; 78 Calleva Research Centre for Evolution and Human Sciences, Magdalen College, Oxford, United Kingdom; 79 Vrije Universiteit Amsterdam, Amsterdam, Netherlands; 80 Department of Psychology, Erzurum Technical University, Erzurum, Turkey; 81 Higher School of Economics, National Research University, Moscow, Russian Federation; 82 Independent Scientist, Krakow, USA; 83 Institute of Psychology, University of Presov, Presov, Slovakia; 84 London Gates Education Group, Riga, Russian Federation; 85 Humanities Department, Icam Toulouse, Toulouse, France; 86 Institute of Linguistics, Shanghai International Studies University, Shanghai, China; 87 Department of Psychology, School of Medical and Life Sciences, Sunway University, Subang Jaya, Malaysia; 88 Green Dock, Hostivice, Czech Republic; 89 English Language and Linguistics, University of Glasgow, Glasgow, Scotland; 90 Matej Bel University in Banská Bystrica, Banská Bystrica, Slovakia; 91 University of Mannheim, Mannheim, Germany; 92 Independent Scientist, Istanbul, Turkey; 93 Department of Psychology, University of Warwick, Warwick, United Kingdom; 94 School of Psychology, Nanjing Normal University, Nanjing, China; 95 Department of Philosophy, Sociology, Education and Applied Psychology, University of Padova, Padova, Italy; 96 SWPS University of Social Sciences and Humanities, Warsaw, Poland; 97 Department of Psychology, Bournemouth University, Poole, United Kingdom; 98 Department of Psychology, Goethe University Frankfurt, Frankfurt, Germany; 99 Institute of Psychology, Dublin, Ireland; 100 Faculty of Humanities and Social Sciences, University of Osijek, Osijek, Croatia; 101 Faculty of Social and Economic Sciences, Comenius University in Bratislava, Bratislava, Slovakia; 102 Centre for Psychological Counselling and Research Norvel, Osijek, Croatia; 103 Institute of Psychology, University of Wrocław, Wrocław, Poland; 104 Lovisenberg Diaconal Hospital, Nic Waals Institute, Oslo, Norway; 105 University of Tehran, Tehran, Iran; 106 Faculty of Media and Communication, Singidunum University, Belgrade, Serbia; 107 Institute of Psychology, University of Hildesheim, Hildesheim, Germany; 108 Lomonosov Moscow State University, Moscow, Russia; 109 Centre of Language Evolution Studies, Nicolaus Copernicus University in Toruń, Toruń, Poland; 110 Psychological Institute of Russian Academy of Education, Moscow, Russia; 111 Charles University, Prague, Czech Republic; 112 Fukuoka Institute of Technology, Fukuoka, Japan; 113 Department of Psychology, Sapienza University of Rome, Rome, Italy; 114 Department of General Psychology, University of Padova, Padova, Italy; 115 University of Vienna, Vienna, Austria; 116 Ankara Medipol University, Altındağ/Ankara, Turkey; 117 Department of Molecular and Cell Biology, University of California Berkeley, Berkeley, USA; 118 Department of Psychology, University of the Philippines Diliman, Quezon City, Philippines; 119 Department of Nursing, Cyprus University of Technology, Limassol, Cyprus; 120 Iscte-Instituto Universitário de Lisboa (Cis-Iul), Lisboa, Portugal; 121 Leeds Beckett University, Leeds, United Kingdom; 122 University of Zadar, Zadar, Croatia; 123 Üsküdar University, İstanbul, Turkey; 124 ELTE - Eotvos Lorand University, Budapest, Hungary; 125 Facultad de Psicología, Fundación Universitaria Konrad Lorenz, Bogotá, Colombia; 126 Global MINDS, University of Limerick, Limerick, Ireland; 127 Kyushu University, Fukuoka, Japan; 128 University of Amsterdam, Amsterdam, Netherlands; 129 Institute for Globally Distributed Open Research and Education (IGDORE), Stockholm, Sweden; 130 Zurich University of Applied Sciences, School of Applied Psychology, Zurich, Switzerland; 131 University of Paris, Paris, France; 132 Sofia University “St. Kliment Ohridsky”, Sofia, Bulgaria; 133 Department of Psychology, University of Konstanz, Konstanz, Germany; 134 Laboratory for Research of Individual Differences, Faculty of Philosophy, University of Belgrade, Belgrade, Serbia; 135 Institute of Psychology, Jagiellonian University, Krakow, Poland; 136 Jose Rizal University, Metro Manila, Philippines; 137 Institute of Psychology, University of Pécs, Pécs, Hungary; 138 Department of Psychology, Faculty for Legal and Business Studies Dr Lazar Vrkatić, Novi Sad, Serbia; 139 INSEAD, Singapore, Singapore; 140 Faculty of Social and Economic Sciences, Institute of European Studies and International Relations, Comenius University, Bratislava, Slovakia; 141 Universidad Latina de Costa Rica, San Pedro, Costa Rica; 142 CLLE, CNRS, Université de Toulouse, Toulouse, France; 143 Department of Psychology, Ben Gurion University, Beersheba, Israel; 144 Department of Experimental Psychology, University of Granada, Granada, Spain; 145 Faculty of Arts, University of Presov, Presov, Slovakia; 146 Institute of Social Sciences CSPS, Slovak Academy of Sciences, Bratislava, Slovakia; 147 Prague University of Economics and Business, Praha, Czechia; 148 Universitas Sebelas Maret, Kota Surakarta, Indonesia; 149 Independent Scientist, Krakow, Poland; 150 Faculty of Humanities and Social Sciences, University of Jyväskylä, Jyväskylä, Finland; 151 University of Oxford, Oxford, UK; 152 Department of Special Education and The Edmond J. Safra Brain Research Center for the Study of Learning Disabilities, University of Haifa, Haifa, Israel; 153 Department of Psychology, University of Zurich, Zurich, Switzerland; 154 University of Osijek, Osijek, Croatia; 155 Independent Researcher, Praha, Czech Republic; 156 Department of Clinical Neuroscience, Karolinska Institutet, Solna, Sweden; 157 Swedish National Data Service, Gothenburg University, Gothenburg, Sweden; 158 Laboratory of Alzheimer’s Neuroimaging and Epidemiology, IRCCS Istituto Centro San Giovanni di Dio Fatebenefratelli, Brescia, Italy; 159 Department of Psychology, Faculty of Integrated Arts & Social Science, Japan Women’s University, Tokyo, Japan; 160 Department of Experimental Psychology, Ghent University, Ghent, Belgium; 161 Södertörn University, Huddinge, Sweden; 162 Independent Scientist, Ankara, Turkey; 163 ISCTE-University Institute of Lisbon, Lisboa, Portugal; 164 Faculty of Philosophy, Institute of Psychology, Jagiellonian University, Warszawa, Poland; 165 Tilburg University, Tilburg, The Netherlands; 166 SWPS University of Social Sciences and Humanities, Sopot, Poland; 167 Karolinska Institute, Stockholm, Sweden; 168 Federal University of Paraíba, Paraiba, Brazil; 169 Department of Psychology, Senshu University, Toyko, Japan; 170 Department of Psychology, Atilim University, Ankara, Turkey; 171 School of Education and Social Sciences, Division of Psychology, University of the West of Scotland, Paisley, Scotland; 172 University of Silesia in Katowice, Katowice, Poland; 173 Sapienza University of Rome, Rome, Slovenia; 174 Center for Research on Cognition and Behavior, SWPS University of Social Sciences and Humanities, Sopot, Poland; 175 CNRS, Université de Lorraine, Nancy, France; 176 The School of Creative Arts Therapies, Faculty of Social Welfare and Health Sciences, University of Haifa, Haifa, Israel; 177 Department of Cognition, Emotion and Methods in Psychology, Faculty of Psychology, University of Vienna, Vienna, Austria; 178 Departamento de Idiomas, Campus Sonora Norte, Tecnológico de Monterrey, Hermosillo, México; 179 Medical and Clinical Psychology, Tilburg University, Tilburg, The Netherlands; 180 Department of Psychology, Education and Child Studies, Erasmus School of Social and Behavioural Sciences, Erasmus University, Rotterdam, The Netherlands; 181 MRC Cognition and Brain Sciences Unit, University of Cambridge, Rotterdam, United Kingdom; 182 Independent Researcher, Istanbul, Turkey; 183 Cognitive Science and Psychology Department, New Bulgarian University, Sofia, Bulgaria; 184 Faculty of Management, University of Warsaw, Warsaw, Poland; 185 Ibn Tofail University (ESEF), Kenitra, Morocco; 186 BETA, Université de Strasbourg, Strasbourg, France; 187 Behavioural Science Institute, Radboud University, Nijmegen, The Netherlands; 188 Tarbiat Modares University, Tehran, Iran; 189 Singapore Management University, Singapore, Singapore; 190 Psychology Department, Florida International University, Miami, USA; 191 Department of Psychology, University of Essex, Colchester, United Kingdom; 192 School of Psychological Sciences, Macquarie University, Sydney, Australia; 193 Chinese University of Hong Kong, Hong Kong S.A.R., China; 194 National and Kapodistrian University of Athens, Athens, Greece; 195 Australian Catholic University, North Sydney, New South Wales Australia; 196 Faculty of Medicine FMUC, Institute of Nuclear Sciences Applied to Health ICNAS, Coimbra Institute for Biomedical Imaging and Translational Research CIBIT, University of Coimbra, Coimbra, Portugal; 197 HSE University, Moscow, Russia; 198 Discipline of Psychology, Centre for Healthy Ageing, Health Futures Institute, Murdoch University, Perth, Australia; 199 University of Bristol, Bristol, United Kingdom; 200 University of Nottingham Malaysia, Semenyih, Malaysia; 201 T A Pai Management Institute, Manipal Academy of Higher Education, Manipal, India; 202 Franklin and Marshall College, Lancaster, USA; 203 Psychology Department, Bar Ilan University, Ramat Gan, Israel; 204 Instituto de Investigaciones Psicológicas (IIPsi), Consejo Nacional de Investigaciones Científicas y Técnicas, Universidad Nacional de Córdoba, Córdoba, Argentina; 205 Department of Psychology, Norwegian University of Science and Technology, Trondheim, Norway; 206 University of Sonora, Sonora, Mexico; 207 Institute of Nuclear Sciences Applied to Health ICNAS, Coimbra Institute for Biomedical Imaging and Translational Research CIBIT, University of Coimbra, Coimbra, Portugal; 208 Witten/Herdecke University, Witten, Germany; 209 Oslo New University College, Oslo, Norway; 210 University of Opole, Institute of Psychology, Opole, Poland; 211 The Pennsylvania State University, State College, USA; 212 Lazaridis School of Business and Economics, Wilfrid Laurier University, Waterloo, Canada; 213 School of Psychology & Neuroscience, University of Glasgow, Glasgow, Scotland UK; 214 Faculty of Law and Business Studies Dr Lazar Vrkatić, Novi Sad, Serbia; 215 School of Psychology, Swansea University, Swansea, United Kingdom; 216 Department of Psychology, University of Crete, Rethymno, Greece; 217 Department for Psychology and Psychotherapy, Witten/Herdecke University, Witten, Germany; 218 University of Edinburgh, Edinburgh, United Kingdom; 219 Neuroscience Program, Dominican University, River Forest, USA; 220 Wittenberg University, Springfield, USA; 221 University of Cyprus, Nicosia, Cyprus; 222 Department of Psychology, University of Maribor, Maribor, Slovenia; 223 University of Delaware, Newark, USA; 224 Discipline of Psychology, Faculty of Health, University of Canberra, Canberra, Australia; 225 Educational Centre “Psychodemia”, Moscow, Russian Federation; 226 Stanford University, Stanford, USA; 227 Psychology Department, Dominican University, River Forest, USA; 228 Samsun University Department of Psychology, Samsun, Turkey; 229 Indiana University Pennsylvania, Indiana, USA; 230 University of Porto, Porto, Portugal; 231 Department of Clinical Psychology, United Arab Emirates University, Al Ain, United Arab Emirates; 232 Division of Psychology and Language Sciences, University College London, London, United Kingdom; 233 Social Science Department, College of Liberal Arts, Technological University of the Philippines, Manila, Philippines; 234 School of Psychology, Arellano University, Metro Manila, Philippines; 235 Jagiellonian University, Krakow, Poland; 236 University of Auckland, Auckland, New Zealand; 237 Nova Southeastern University, Broward County, USA; 238 Virginia Commonwealth University, Richmond, USA; 239 Department of Psychology, University of Minnesota, Minneapolis, USA; 240 Laboratoire Parisien de Psychologie Sociale, Université Paris Nanterre, Nanterre, France; 241 Grupo de Investigación en Comunicación y Salud, Instituto de Investigación Científica, Universidad de Lima, Paris, Peru; 242 Open University, Milton Keynes, United Kingdom; 243 London School of Economics and Political Science, London, United Kingdom; 244 School of Health and Behavioural Sciences, University of the Sunshine Coast, Petrie, QLD, Petrie, Australia; 245 Department of Psychology, Indiana University of Pennsylvania, Indiana, USA; 246 Division of Community Behavioral Health, Department of Psychiatry and Behavioral Sciences, University of New Mexico Health Sciences Center, Albuquerque, USA; 247 University of Florida, Gainsville, USA; 248 Cal Poly Humboldt, Humboldt, USA; 249 Department of Experimental and Applied Psychology, Vrije Universiteit Amsterdam, Amsterdam, The Netherlands; 250 Department of Psychology, Government College University, Lahore, Pakistan; 251 Universidad del Desarrollo, Concepcion, Chile; 252 Department of Psychology, University of Denver, Denver, USA; 253 Yale School of Public Health, Department of Chronic Disease Epidemiology, New Haven, USA; 254 Psychology Department, Universidad de los Andes, Bogota, Colombia; 255 Institute of Psychology, University of Wroclaw, Wrocław, Poland; 256 Northern Illinois University, Dekalb, USA; 257 University of Alabama, Tuscaloosa, USA; 258 Universidad de Sonora, Hermosillo, México; 259 Universidade de Brasília, Brasília, Brasil; 260 School of Behavioural and Health Sciences, Australian Catholic University, New South Wales, Australia; 261 Facultad de Psicología, Universidad del Desarrollo, Concepcion, Chile; 262 Faculty of Psychology, University of Desarrollo, Desarrollo, Chile; 263 NTU Psychology, Nottingham Trent University, Nottingham, United Kingdom; 264 Faculty of Higher Studies Iztacala, National Autonomous University of Mexico, Mexico City, Mexico; 265 Department of Psychology, Sewanee: The University of the South, Sewanee, USA; 266 University of Aberdeen, Aberdeen, Scotland; 267 University of Western Australia, Crawley, Australia; 268 University of Tabriz, Tabriz, Iran; 269 Environment Centre, Charles University, Prague, Czech Republic; 270 Saint Joseph’s University, Philadelphia, USA; 271 State University of New York at Fredonia, Fredonia, USA; 272 School of Psychology, University of East London, London, UK; 273 Max Planck Institute for Research on Collective Goods, Bonn, Germany; 274 Department of Psychology, Alex Ekwueme Federal University, Ndufu-Alike, Nigeria; 275 Manchester Metropolitan University, Manchester, United Kingdom; 276 Department of Psychology, Daulat Ram College, University of Delhi, Delhi, India; 277 Faculty of Philosophy, University of Belgrade, Belgrade, Serbia; 278 School of Education and Psychology, University of Navarra, Pamplona, Spain; 279 Psychological Processes in Education and Health Group, School of Education and Psychology, University of Navarra, Pamplona, Spain; 280 Faculty of Economics, Maria Curie-Sklodowska University, Lublin, Poland; 281 Facultad de Psicología, Universidad San Sebastián, Valdivia, Chile; 282 Department of Marketing, Fox School of Business, Temple University, Philadelphia, USA; 283 IDN Being Human Lab, University of Wrocław, Wrocław, Poland; 284 School of Anthropology & Museum Ethnography, University of Oxford, Oxford, United Kingdom; 285 Department of Population and Quantitative Health Sciences, Case Western Reserve University, Cleveland, USA; 286 Department of Law and Criminology, Royal Holloway, University of London, London, UK; 287 University of Wisconsin-Stout, Menomonie, USA; 288 University of Hong Kong, Hong Kong S.A.R., China; 289 Western Kentucky University, Bowling Green, USA; 290 Fo Guang University, Jiaoxi, Taiwan; 291 Grenoble Ecole de Management, Grenoble, France; 292 Middlebury College, Middlebury, USA; 293 Michigan State University, East Lansing, USA; 294 Purdue University, West Lafayette, USA; 295 Department of Organization and Human Resources, UQAM, Montreal, Canada; 296 Psychology Department, Ithaca College, Ithaca, USA; 297 Institute of Advanced Studies in Humanities and Social Sciences, Beijing Normal University, Zhuhai, China; 298 Department of Psychology, University of Hong Kong, Hong Kong S.A.R., China; 299 Department of Social and Work Psychology, University of Brasília, Brasília, Brazil; 300 University of Galway, Galway, Ireland; 301 Black Dog Institute, UNSW Sydney, Sydney, Australia; 302 Emotional Health for All Foundation, Jakarta, Indonesia; 303 University of Wuppertal, Wuppertal, Germany

**Keywords:** Diseases, Research data

## Abstract

In response to the COVID-19 pandemic, the Psychological Science Accelerator coordinated three large-scale psychological studies to examine the effects of loss-gain framing, cognitive reappraisals, and autonomy framing manipulations on behavioral intentions and affective measures. The data collected (April to October 2020) included specific measures for each experimental study, a general questionnaire examining health prevention behaviors and COVID-19 experience, geographical and cultural context characterization, and demographic information for each participant. Each participant started the study with the same general questions and then was randomized to complete either one longer experiment or two shorter experiments. Data were provided by 73,223 participants with varying completion rates. Participants completed the survey from 111 geopolitical regions in 44 unique languages/dialects. The anonymized dataset described here is provided in both raw and processed formats to facilitate re-use and further analyses. The dataset offers secondary analytic opportunities to explore coping, framing, and self-determination across a diverse, global sample obtained at the onset of the COVID-19 pandemic, which can be merged with other time-sampled or geographic data.

## Abstract (summary)

**Table:** 

Measurement(s)	COVID-19 Protocols, Restrictions • Health Behaviors • Personality and Behavioral Change, CTCAE • Emotion • Message Framing • Self-Determination
Technology Type(s)	Survey • Experiment Design Type
Factor Type(s)	COVID-19 Protocols, Restrictions • Health Behaviors • Loss-Gain Framing • Cognitive Reappraisal • Self-Determination Messaging
Sample Characteristic - Organism	Homo
Sample Characteristic - Environment	Daily Life
Sample Characteristic - Location	North America • South America • Africa • Australia • Europe • United Kingdom • Asia

## Background & Summary

**Fig. 1:**
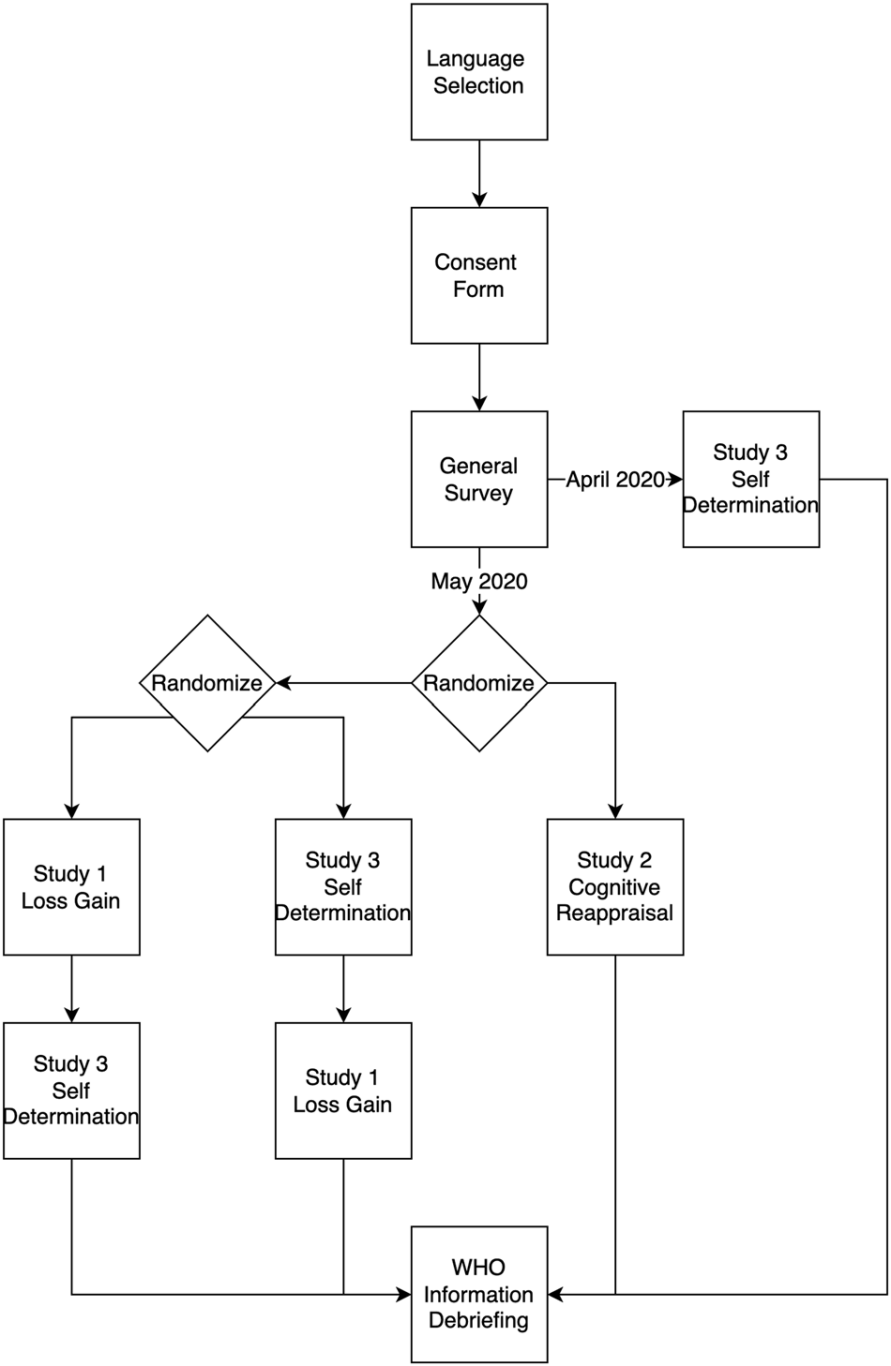
Survey flow for the PSACR project. As shown in Fig. 2, participants were given one path through the study determined by the date they completed the study and randomization factors.

**Fig. 2:**
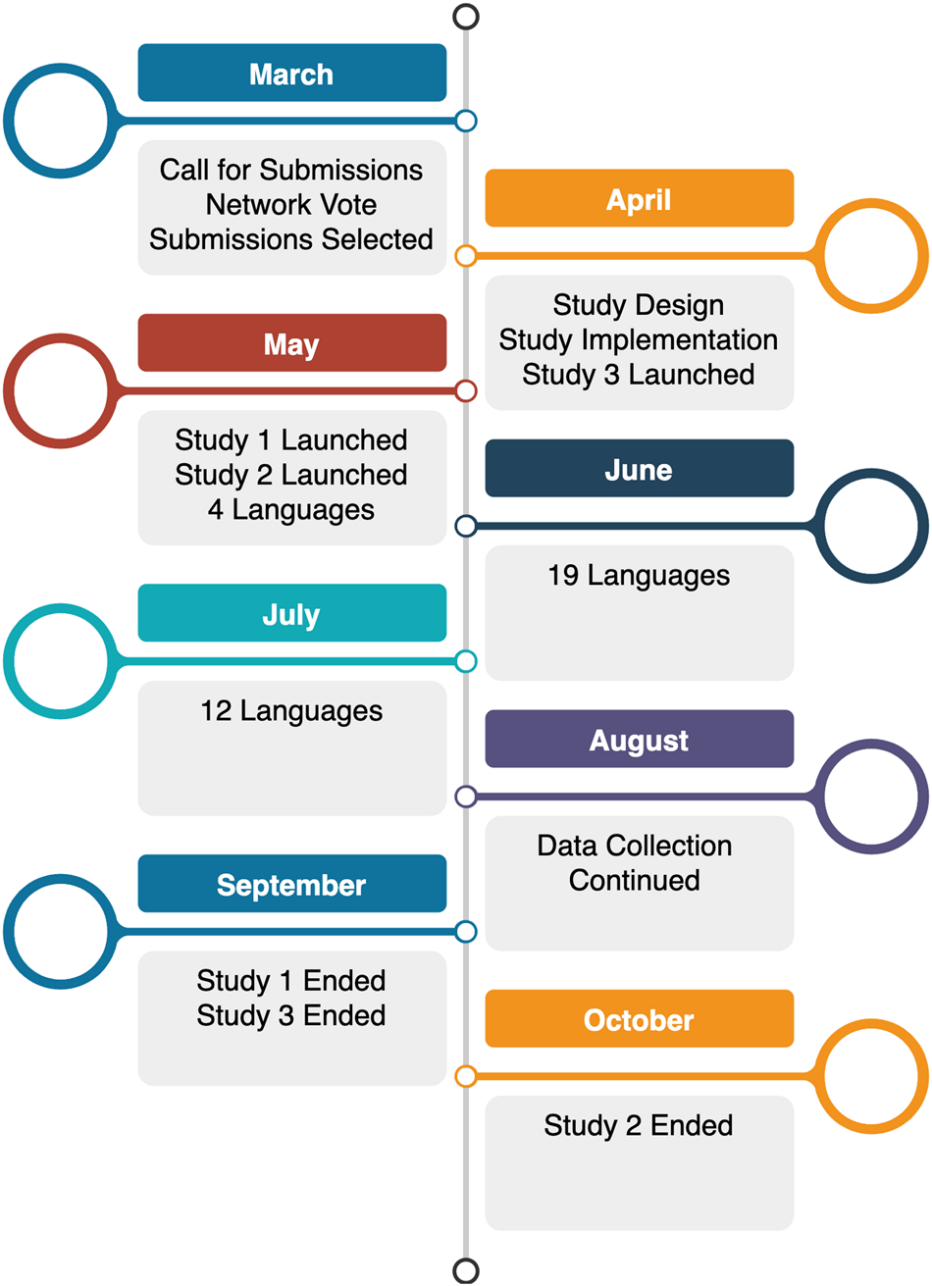
Timeline for the PSACR project in 2020. In April, only the English version of the study was available for participants. In May, Hungarian, Dutch, Polish, and Portuguese were added to the study. In the next month, French, Macedonian, Swedish, Spanish, Farsi, Norwegian, Russian, Turkish, Bulgarian, Urdu, Czech, Greek, Italian, Japanese, Slovak, Arabic, Hebrew, Filipino, and Korean were launched. In July, Croatian, German, Yoruba, Armenian, Chinese, Serbian, Finnish, Romanian, Uzbek, Bengali, Slovenian, and Hebrew were included. Languages were generally launched with its dialect variants (e.g., Dutch and Dutch-Belgian).

In 2020, the rise of the COVID-19 pandemic presented enormous challenges to people’s health and well-being. In response to this challenge, in March 2020, the Psychological Science Accelerator (PSA)^1^ announced a call for studies focusing on applied research to answer questions on how to reduce the negative emotional and behavioral impacts of the pandemic, the PSA COVID-Rapid (PSACR) Project. The PSA is a global collaborative network of over 1,000 members across 80+ countries/geopolitical locations that develop large, “big-team science” projects. Three research studies were selected to pursue, paired with a general survey about health behaviors, COVID-19 experiences, and demographic information (https://osf.io/x976j). The dataset described here represents three studies on the psychology of message communication: Study (1) how framing affects health communication messages using gain-versus-loss framing^2^; Study (2) cognitive reappraisal^3^; and Study (3) how self-determination theory can inform health messaging for social distancing uptake^4^. Participants either completed Loss-Gain Framing (Study 1) and Self-Determination (Study 3) (order counterbalanced), only the Self-Determination (Study 3) or Cognitive Reappraisal (Study 2) (see Figs. 1 and 2).

### Loss-gain framing (Study 1)

In the first study, participants read health excerpts that framed these messages behaviors as gains or losses, and we subsequently measured participants’ intentions to follow guidelines to prevent COVID-19 transmission, attitudes towards COVID-19 prevention policies, propensity to seek additional information about COVID-19, and anxiety (using both self-report and behavioral measures)^2^. This study was designed to examine the role of message framing in influencing compliance with the pandemic recommendations using gain-versus-loss framing. The gain-framed messages in this study highlighted the usefulness of compliance with the messages (e.g., there is so much to gain. If you practice these four steps, you can protect yourself and others), whereas the loss-framed messages spotlighted the negative effects of ignoring the recommendations (e.g., there is so much to lose. If you do not practice these four steps, you can endanger yourself and others)^5^. Research on the impact of message framing on emotion and behavior is critical^6^, and previous studies suggest that loss-framing elicits negative emotion^7^. This study extended previous research to measure the impact of framing on anxiety.

#### Summary of study findings

Loss-framed messages (as compared to gain-framed) increased participants’ self-reported anxiety. On the contrary, there were no meaningful differences between gain framing and loss framing in effects on 1) behavioral intentions to follow COVID-19 prevention guidelines, (2) attitudes toward COVID-19 prevention policies, and 3) whether participants chose to seek more information about COVID-19. Crucially, most of these results were relatively consistent across 84 countries.

### Cognitive reappraisal (Study 2)

The second study focused on emotion regulation through cognitive reappraisal^3^. The COVID-19 pandemic has been shown to decrease positive emotions and psychological health and increase negative emotions^8–10^. These emotional changes could potentially be mitigated by emotion regulation strategies. Cognitive reappraisal is one such strategy that encourages changes in the way one thinks about a situation in order to change the way one feels^11^, and this study specifically used reconstrual and repurposing as reappraisal methods. Previous research has shown that participants engaging in cognitive reappraisal can increase resilience in a simple and effective manner^12^, especially in comparison to other emotion strategies, such as emotion suppression^13^. Study 2 examined the effectiveness of reappraisal strategies to reduce negative emotions and increase positive ones regarding COVID-19 situations. Moreover, we explored whether cognitive reappraisals influence health behavioral intentions.

#### Summary of study findings

The two variations of reappraisal strategies tested in this study (reconstrual and repurposing) both reduced negative emotions and increased positive emotions related to COVID-19 situations compared to control conditions. Neither strategy affected health behavior intentions related to stay-at-home behaviors and hand washing behaviors. The two strategies had similar effects to one another.

### Self-determination (Study 3)

The third study examined motivations/intentions to participate in social distancing using self-determination theory as a framework to design messages that either induced individual autonomy through supportive messaging or pressured individuals through controlling messaging^14,15^. Autonomy-supportive communication styles include information on perspective-taking, the rationale for changing behavior, and engaging an individual’s autonomy of choice in a scenario^16^. Controlling communication styles use shame and blame to induce a change in behavior^17^. In general, autonomy-supportive messages have been shown to increase behavior change^18^, whereas controlling messages often lead to behaviors that are the opposite of what was intended^19,20^. Study 3 examined the effects of autonomy-supportive versus controlling messages about social distancing on the quality of motivation, feelings of defiance, and behavioral intentions to engage in social distancing.

#### Summary of study findings

The controlling message increased controlled motivation for social distancing, a relatively ineffective form of motivation concerning avoiding shame and social consequences, relative to the control group who received no message. On the other hand, the autonomy-supportive message decreased feelings of defiance compared with the controlling message. Unexpectedly, neither autonomy-supportive or controlling messages influenced behavioral intentions, though existing motivations did, with autonomous motivation predicting greater long-term intentions, and controlled motivation predicting fewer long-term intentions to social distance.

Here, we present the aggregated data from all studies and from a health behavior survey assessing demographics and COVID-19 experience and local restrictions^21^. These data can be further merged with corresponding data using time and/or location information, potentially to track responses in tandem with COVID-19 rates (https://data.humdata.org/dataset/oxford-covid-19-government-response-tracker), vaccinations^22^, hospitalizations (https://ourworldindata.org/coronavirus-data-explorer), or other psychological variables (e.g., anxiety, depression; https://www.nih.gov/news-events/news-releases/all-us-research-program-launches-covid-19-research-initiatives). The dataset provides an array of untapped insights into human emotion, motivation, persuasion, and other topics that can be unlocked through secondary analyses (e.g., regional moderator analyses).

## Methods

### Survey flow

The PSACR project consisted of a general survey (i.e., consisting of health behaviors, COVID-19 information, and demographics) and the three studies described above (see the survey flow in Fig. 1). Data were collected online, and participants were able to complete the study from any internet-enabled device. First, participants were shown a landing page to select their language or regional variation of a language, with 44 languages included as options. Participants then read an informed consent form to familiarize themselves with the general objectives of the study and indicate their consent to participate in the study (yes/no choice). The entire study was approved by the Institutional Review Board at Ashland University as the main research hub, and local approval was obtained from other research labs depending on individual institution requirements.

The participants were then shown a general survey, COVID-19 experience, and demographics survey designed by the PSACR admin team (described below). Participants were then randomly invited to complete either (a) Cognitive Reappraisal (Study 2), or (b) both the Loss-Gain Framing (Study 1) and Self-Determination (Study 3) studies in random order. However, a small portion of participants received only Study 3 (Self-Determination), as this study was deployed before the other two studies were ready (see Fig. 2 for a timeline of the project). Creating two survey flows (Study 1 and 3 versus Study 2) kept participant completion time to approximately 20–30 minutes across the entire survey as Study 2 took longer to complete than Study 1 and 3. Data collection for Loss-Gain Framing (Study 1) and Self-Determination (Study 3) ended before Cognitive Reappraisal (Study 2) for which data collection was continued to achieve the *a priori* determined sample size. A timeline for the different PSACR studies onsets and offsets is presented in Fig. 2. After completing the studies, participants were shown a debriefing about the studies that included information about the pandemic and World Health Organization (WHO) recommendations. All translations and materials can be found at https://osf.io/gvw56/ and at https://osf.io/s4hj2/.

### Translation

The translation team consisted of one lead coordinator, two assistant coordinators, one language specific coordinator, and translators within each language. The lead coordinator, along with the assistant coordinators, oversaw all languages organizing and connecting information between the study teams, translation teams, and implementation teams. The language coordinator worked with the individual translators for their target language, and approximately 268 individuals helped achieve the translations for this study. The translation process included three stages: forward translation, backward translation, and cultural adjustment. During forward translation, at least two translators worked together to translate each study from English to each target language. Separately, two translators then reversed this process by backward translating from each target language to English. Between these two stages, all inconsistencies were eliminated by discussion between team members. Last, the translated materials were sent to separate individuals for cultural adjustment. Cultural adjustment included wording tweaks for understanding within that culture. For example, several of the educational levels were modified based on the education system in the area that generally spoke the target language (see https://osf.io/ca3ks for a review of the translation process).

### Study deployment

The study was implemented online using *formr* survey software^23^ which enabled complex randomization and study tracking during the life of the project. The study can be reproduced using the Excel files for each language and the overall survey flow **psacr-pool.json** file (https://osf.io/643aw/) provided in the **formr** folder in the Open Science Framework (OSF) repository. *formr* software allows a researcher to import a survey flow through .json formatted files, and the exact questions and study design can be imported using Excel or Google Sheets files. Within each language folder, we have provided the consent form, the general survey, the Loss-Gain Framing survey (Study 1), the Cognitive Reappraisal survey (Study 2), the Self-Determination survey (Study 3), and the final WHO and debriefing information. Each language setup uses the same survey flow and therefore can be recreated using the provided .json survey flow and the relevant Excel files.

### General survey

The general survey was designed to gather information about the participants’ experiences with COVID-19 protocols and restrictions in their lives as well as demographic information. Participants first reported their health behaviors in response to the pandemic by indicating how many times they had left their homes in the past week, the reasons for leaving their home, their mask usage, hand washing, and coughing/sneezing actions. The next section covered COVID-19 restrictions and government responses, including current restrictions, ability to manage restrictions, and trust and satisfaction in government activities. Participants then indicated if they had been tested for COVID-19, were self-isolating for symptoms, their confidence about understanding and preventing COVID-19, and their worry over their physical and emotional well-being. The last section of the general survey covered participant demographics (age, gender, education, geopolitical region, nationality, state of residence for U.S. participants, and type of community), questions about how the participant was recruited into the survey, and questions about their household (number of members, socioeconomic status, and health conditions).

### Loss-gain framing (Study 1)

In this study, each participant was first presented with an overall description of the task, which was to express their opinions on various recommendations for mitigating COVID-19. Participants were randomized into two framing conditions about the steps one can take to meet COVID-19 guidelines. These two conditions were framed in either a gain perspective (“You have so much to gain by practicing these steps”) or a loss perspective (“You have so much to lose by not following these steps”). Within the loss- and gain-framed conditions, the messages were written in three different ways, and participants were randomly assigned which version of the message to view. Participants then rated the likelihood to comply with these recommendations while the instructions were presented on the page. Next, they rated their feelings on government policies related to health, rated their emotions (i.e., anger, anxiety, fear) when considering these policies, and indicated if they wanted to learn more about WHO guidelines. Finally, participants were asked to complete a manipulation check about the information they had read to ensure they had paid attention during the study.

### Cognitive reappraisal (Study 2)

This study focused on determining if a cognitive reappraisal strategy would change the emotional responses to photos related to COVID-19. First, participants rated their emotions they were feeling “right now.” For positive emotions, they indicated how hopeful, loved, peaceful, understood, and cared for they felt. For negative emotions, they indicated how annoyed, sad, angry, stressed, left out, and much hate they felt. These emotions were randomly presented such that each person saw positive and negative emotion questions interspersed, beginning either with positive (i.e., positive, negative, positive, negative, etc.) or negative. The order of the questions was also randomized across participants.

At this point in the study, participants were randomized into one of four conditions: (1) reflecting: reflecting upon your thoughts and feelings about any situation can lead to different emotional responses; (2) rethinking: different ways of interpreting or thinking about any situation can lead to different emotions; (3) refocusing: finding something good in even the most challenging situations can lead to different emotional responses; and (4) a control condition that suggested participants respond as they naturally would. Participants were provided with instructions on how to implement these appraisals. Consequently, participants had four practice trials where each time they viewed a picture, rated their current affect/mood, and wrote a few sentences about how they implemented the appraisal with respect to the picture.

After completion of the practice trials, participants rated ten COVID-19 related pictures (e.g., person on a stretcher being taken out of an ambulance, grieving/worried relatives, etc.) followed by questions about how positive and negative they felt viewing the pictures. The pictures were selected by lead team of Study 2 by searching major news sources across Asia, Europe, and North America. These pictures were rated by the lead team on sadness/anxiety and whether they should use the pictures. The final selection of pictures received higher-than-average scores (on a 1–7 Likert scale), for both ratings. This section was followed by the original emotion questions presented at the beginning of the study, in the same randomized order as they had been previously seen. These questions were then adjusted to determine anticipated emotions (i.e., to what extent will you feel sad). Participants next answered questions about their positive habits, such as exercising, and negative habits, such as drinking and smoking. Last, participants were given a series of attention check questions to identify inattentive responding, along with a final question about their current emotional state relative to their emotional state at the beginning of the study.

### Self-determination (Study 3)

This study examined the COVID-19 recommendations for social-distancing across three randomly assigned conditions: (1) an autonomy-supportive message promoting personal agency and reflective choices, (2) a controlling message that was forceful and shaming, and 3) no message about social distancing. Each participant rated their current adherence with social distancing before receiving these messages. After the messages, participants rated their motivations to engage in social distancing, and how the messaging about social distancing made them feel. They then re-rated the original items about their social distancing intentions for the next week (i.e., how often will you see friends in the next week) and the next six months. At the end of the study, participants completed manipulation check items to ensure messages were experienced as supportive vs. pressuring.

### Data processing

The complete data processing scripts from the study, along with annotations, can be found at https://osf.io/gvw56/. During the data collection phase of the studies, we tracked several indicators such as the current participant counts, timing, and other important factors in the study (e.g., the number of people in each language and group). The code for this tracking is presented in https://osf.io/uzqdr/, but those summary data were collected only for monitoring purposes during the study. The following data processing steps were taken on the raw data, and the final output can be found in the **raw_data** folder in the OSF repository. The raw data are stored separately by month in compressed zip files due to their large size and file storage limits. The processing steps included:

1. Eliminate duplicate rows. We collected data across multiple servers, and sometimes, the server posted the same participant data twice.
2. Creation of unique identifiers for each participant. One language of the study (Swedish) briefly did not include the appropriate code to enable creating unique identifiers. Therefore, these data were matched using other information embedded in the surveys and the unique identifier was filled in for these participants.
3. Removal of pilot data. We removed pilot testing responses from the dataset. These responses were identified by the start dates and times for each language separately.
4. Participant completion codes (e.g., unique subject identifiers created for reporting completion at the end of the study) were extracted from the raw data for researchers to check if their participants had completed the study.
5. Participant personal information (i.e., information that could be used to identify the participant, information for creating completion codes, emails or phone numbers for lottery participants) was excluded before uploading data into the **raw_data** folder.

Next, we transformed the raw data set to facilitate reuse of the data. The code for this stage can be found at https://osf.io/shd9a/. The following steps were performed to create a processed dataset, and the output from this data curation can be found in the **processed_data** folder online.

1. The information presented on the screen to the participant was included as a new column matching each item label from the *formr* worksheets. These labels were added as new columns in English and the language the study was displayed in.
2. The answer choice from a participant is included in numeric format (e.g., 1 = *Strongly Agree* to 5 = *Strongly Disagree*), and therefore, we included a new column with the original labels of the answer choice for items that had text labels. These labels are in English, even if the study was presented in a different language.
3. We created an overall participant file that has relevant information for participants across the entire study. This data file is described below.
4. We separated the data from each of the embedded studies (General Survey, Loss Gain, etc. described above) into independent data files, and these files are described below.
5. For the Cognitive Reappraisal study, we added a column that identified the exact items shown to participants. The original labels contain markdown and *R* code that generated the random order of items for each participant. The label column includes that code but can be difficult to interpret. Therefore, we recreated the order seen by each participant and merged that information into the Cognitive Reappraisal study file only.

### Exclusion criteria

Each of the three individual studies used different exclusion criteria for their analyses (see technical validation description below). For the dataset described in this manuscript, no exclusion criteria were employed other than data curation described above. Therefore, the dataset includes all answers from any person that opened the survey, regardless of completion times or missing data.

## Data Records

In this section, we describe the contents of the **processed_data** folder created from the data processing steps described above. All data is stored on OSF^21^. The **raw_data** folder contains files in a similar structure, which should be interpretable from the descriptions provided below. The main difference in these files is that the processed data include additional columns to disambiguate the information contained in each row.

### Global participant data

**Fig. 3:**
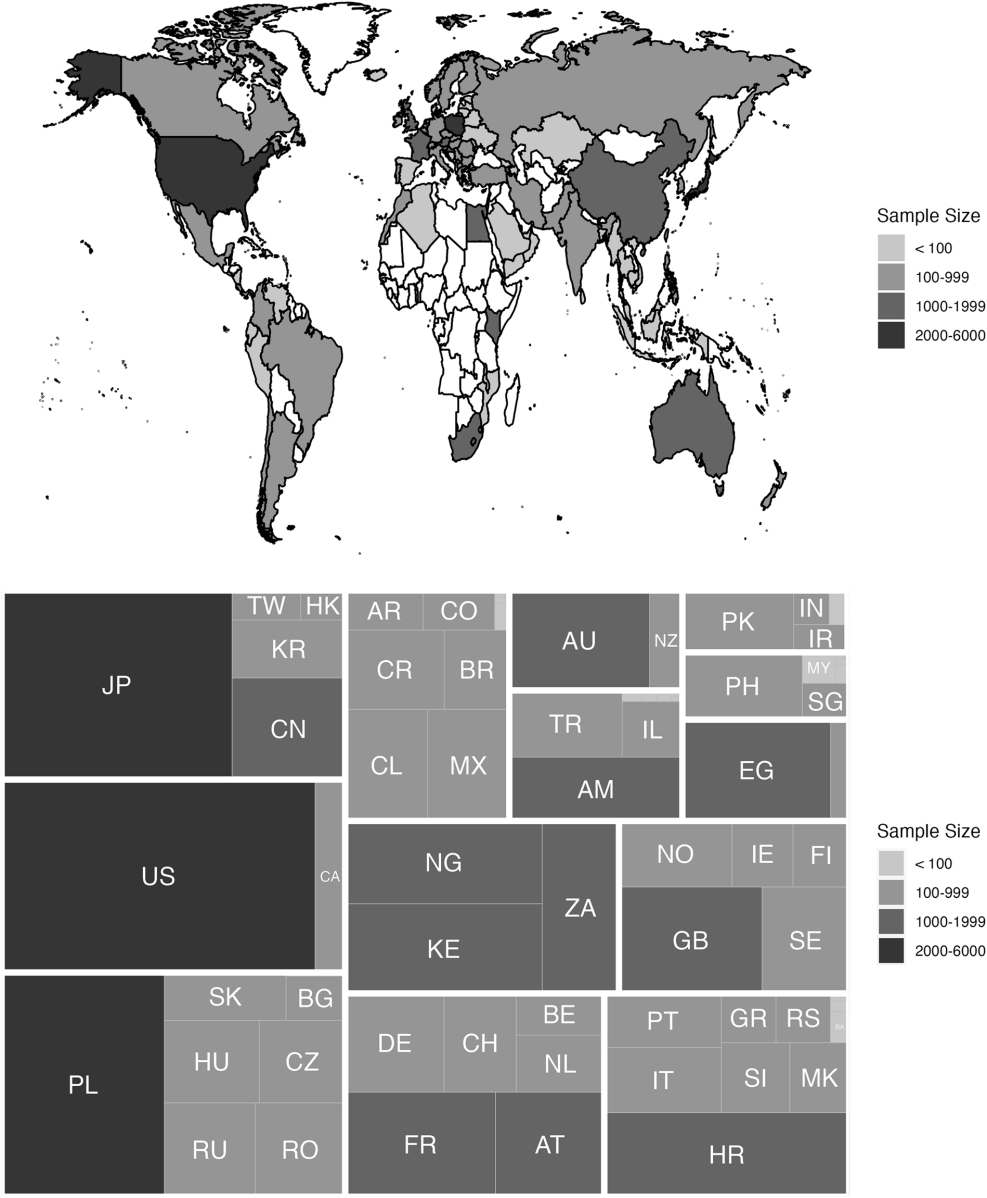
The top panel includes a map of the countries/geopolitical locations from which data was collected for the PSACR project with corresponding sample size. The bottom panel includes a treemap of sample size by geopolitical region, and these values are grouped by UN subregion: Eastern Europe, Northern America, Eastern Asia, Western Europe, Southern Europe, Sub-Saharan Africa, Northern Europe, Latin American and the Caribbean, Western Asia, Australia and New Zealand, South-Eastern Asia, and Southern Asia (listed here largest to smallest). Cell size depicts relative sample size for each sub-region to the whole sample and within groups relative sample size.

The overall participant data consist of one participant information file and two dictionary files that detail the hand-coded processing of a free text response. The participant information file (**participants.csv**) is a comma separated text file that contains overall participant information and can be accessed at https://osf.io/rjgwh/. The information for each column of the participant file is found in Supplemental Table 1. The file included the geopolitical region from which the participant indicated they had taken the survey, along with the state (only for U.S. participants). This information was a free text response in the original survey. The **country_dictionary.xlsx** (https://osf.io/8zv42/) and **state_dictionary.xlsx** (https://osf.io/5xbce/) were used to merge geopolitical information from the overall general survey into this file. These files are in Excel format with two tabs. Each text answer was examined by two individuals and coded into geopolitical region codes and U.S. state names. In cases of disagreement, a third person arbitrated that decision. The first tab of each document includes the final chosen answer, and the second tab includes all coder responses for transparency. Supplemental Tables 2 and 3 include the metadata for each column. Figure 3 demonstrates the geopolitical region data and corresponding sample sizes from this processing.

### Individual study data

The data are in long format wherein each line represents a single item that was shown to the participant, including submit buttons, notes, and hidden item information. All four data files have the same format (Supplemental Table 4). The general survey data can be found at https://osf.io/37uca/ (**general_data.csv.zip**), the Loss-Gain Framing study at https://osf.io/ctsrk/ (**study1_data.csv.zip**), the Cognitive Reappraisal study at https://osf.io/bec2f/ (**study2_data.csv.zip**), and the Self-Determination study at https://osf.io/aqkjh/ (**study3_data.csv.zip**). Each file is in comma separated text format, which was then compressed into a zip archive to fit within space limitations for OSF. As noted in Supplemental Table 4, the Cognitive Reappraisal study has one extra column of data due to the specific randomization involved in the study.

Table 1 includes information about completion rates and demographic measures for each study. The number of participants who started the survey is first reported. Each participant started with the general survey indicating a large portion of the participants generally opened the link to the study and then declined to continue (i.e., 73,223 participants opened the link while only 54,952 started the general survey). The number of participants who completed the last item in each section is also reported. In the next row, we present the number of participants who completed most of the items (>95%) on the survey as a secondary measure of the amount of usable data within each area. Depending on the secondary interest variables, more data may be available for each study. The number of languages and geopolitical regions represented in each study are found next in the table and the overall demographics can be found in Fig. 3. The gender breakdown is provided for each study, and most participants identified as female. Last, we provide information for technical validation described below.

**Table 1:** Completion and Demographic Information.

	General Study	Loss-Gain Framing Study 1	Cognitive Reappraisal Study 2	Self-Determination Study 3
Number Started Survey	54952	17682	27979	20033^a^
Number Completed Last Item	48434^b^	16775^c^	21425	18806
Number Completed Most Items	48182	16799	21447^d^	18999
Number Languages	44	44	43	44
Number Geopolitical Regions	110	89	89	90
Gender				
Female	30226	10300	13543	11602
Male	17277	6185	7575	6864
Prefer Not to Say/Other	557	187	219	223
Missing	374	103	88	117
*M* Minutes Completion	7.51	4.19	20.41	6.37
*SD* Minutes Completion	5.27	4.03	10.71	4.94
Manipulation Check Passed	NA	12049	13859^d^	NA

*Note*. All items starting with Number Languages are calculated based on participants who completed the last item in the study.

^a^The published version of this study added a secondary dataset collected by the lead authors. That data is not included in our publication or these sample sizes.

^b^When more participants complete the last item versus all items, this generally occurred because participants skipped many items or pages of the survey.

^c^When more participants complete most items versus the last time, this generally occurred when people left the survey early or skipped the last page (either on purpose or because they clicked Continue twice too quickly).

^d^This study used two exclusions: participants had to answer at least one manipulation check item correctly (here, we present the number who answered both correctly), and they had to complete at least 50% of the study items (here, we present people who completed most items).

## Technical Validation

As participants entered the survey, they were randomized into one of two combinations of studies (Cognitive Reappraisal only or Loss-Gain Framing and Self-Determination combined). Within each of these studies, the experimental group manipulations were randomly assigned. Last, when noted above, study items were randomly ordered. The participants were blind to their conditions within the study. The technical programmers and study lead investigators had knowledge of the study conditions, but these were not known to most of the data collection teams. Samples were recruited from participant pools, paid participant websites, and social media. Research teams had no control over the allocation of participants to conditions. Samples were tracked through an online Shiny app (i.e., an *R* statistical software that creates online interactive applications^24^) that displayed the total number of participants, separated by language and condition, to ensure appropriate randomization during the study deployment.

As shown in Table 1, the timing of the study completion may be used to ensure data quality by excluding participants who completed the study too quickly (as defined by a secondary analysis team). Each individual study also included manipulation checks to determine participant attentiveness. In the Loss-Gain Framing study, each participant was asked to indicate which framing message they had seen, and the number of participants indicated in Table 1 represents those who chose the correct answer based on their group assignment. The Cognitive-Reappraisal study had two manipulation check questions: participants had to pick a picture they had not previously seen and had to indicate the instructions they had been given to appraise those pictures. The values in Table 1 represent those who answered both questions correctly.

The Self-Determination study did not use a manipulation check with a “correct” answer, but rather examined if their manipulation changed the responses to questions presented at the end of the study (1 *Strongly Disagree* to 7 *Strongly Agree*). The autonomy group (*M* = 4.11, *SD* = 1.70) rated the instructions as providing more choices than the controlling message group (*M* = 3.57, *SD* = 2.01, *d* = 0.29) and the no message group (*M* = 3.75, *SD* = 1.82, *d* = 0.20), while the controlling message group rated the messages lower than the no message group (*d* = 0.10). A second question asked participants to rate the messages seen as trying to pressure people. The controlling messages group rated the item the highest (*M* = 3.30, *SD* = 2.07) in comparison to the autonomy message group (*M* = 2.62, *SD* = 1.91, *d* = 0.34) and the no message group (*M* = 2.88, *SD* = 1.96, *d* = 0.21). The no message group rated this item as higher than the autonomy group, *d* = 0.14. While these effect sizes are small, they indicate a pattern of responses that support participants’ responding to the manipulations presented for their group.

## Supplementary information

Supplemental Table 4

Supplemental Table 1

Supplemental Table 2

Supplemental Table 3

## Data Availability

All code can be found at https://osf.io/gvw56/.
